# Genetic variants in mannose receptor gene (*MRC1*) confer susceptibility to increased risk of sarcoidosis

**DOI:** 10.1186/1471-2350-11-151

**Published:** 2010-10-28

**Authors:** Takeshi Hattori, Satoshi Konno, Ayumu Takahashi, Akira Isada, Kaoruko Shimizu, Kenichi Shimizu, Natsuko Taniguchi, Peisong Gao, Etsuro Yamaguchi, Nobuyuki Hizawa, Shau-Ku Huang, Masaharu Nishimura

**Affiliations:** 1 First Department of Medicine, Hokkaido University School of Medicine, Sapporo, Hokkaido, Japan; 2 Johns Hopkins Asthma & Allergy Center, Johns Hopkins University School of Medicine, Baltimore, MD, USA; 3 Division of Respiratory Medicine and Allergology, Aichi Medical University, Aichi, Japan; 4 Department of Pulmonary Medicine, Institute of Clinical Medicine, Graduate School of Comprehensive Human Sciences and University Hospital, University of Tsukuba, Tsukuba, Ibaraki, Japan

## Abstract

**Background:**

Mannose receptor (MR) is a member of the C-type lectin receptor family involved in pathogen molecular-pattern recognition and thought to be critical in shaping host immune response. The aim of this study was to investigate potential associations of genetic variants in the *MRC1 *gene with sarcoidosis.

**Methods:**

Nine single nucleotide polymorphisms (SNPs), encompassing the *MRC1 *gene, were genotyped in a total of 605 Japanese consisting of 181 sarcoidosis patients and 424 healthy controls.

**Results:**

Suggestive evidence of association between rs691005 SNP and risk of sarcoidosis was observed independent of sex and age in a recessive model (*P *= 0.001).

**Conclusions:**

These results suggest that *MRC1 *is an important candidate gene for sarcoidosis. This is the first study to imply that genetic variants in *MRC1*, a major member of the C-type lectin, contribute to the development of sarcoidosis.

## Background

Sarcoidosis is a multi-organ inflammatory disease with exaggerated cellular immune activity that leads to formation of non-caseating granulomas in the affected organs. Although the causes of sarcoidosis remain unclear, several lines of evidence support the idea that sarcoidosis results from exposure of genetically susceptible individuals to unknown environmental triggers [1-9]. Amomg this evidence, environmental pathogens such as *Mycobacterium *and *Propionibacterium *species have been suggested to play roles in the pathogenesis of sarcoidosis [7-9]. Recent insights into the complex mechanisms underlying human innate immunity suggest that genetic variability in the genes encoding immune system components plays a role in the development of chronic inflammatory diseases. In particular, the association between sarcoidosis and genetic variants of several pattern recognition receptors (PRRs), such as Toll-like receptors (TLRs), have been extensively analyzed [10-12]. Although TLRs are an important system for microbial sensing, they are not the only PRRs with this function. At the cell-surface, C-type lectin-like molecules, such as the mannose receptor and the β-glucan receptors, also participate in the binding and uptake of microbial components [13,14]. Components of bacteria and viruses that gain entry into the cytoplasm are recognized by cytosolic receptors, through which they induce cytokine production and cell activation [15,16]. Recent studies have shown the genetic association between sarcoidosis and non-TLRs [11,17]. Mannose receptor (MR) is a member of the pattern-recognition C-type lectin receptor (CLR) family, which plays an important role in innate immunity. MR is predominantly present on macrophages and dendritic cells and recognizes glycan structures containing mannose, fucose and N-acetylglucosamine, which are commonly found on the cell walls of pathogenic micro-organisms such as mycobacteria, fungus, parasites and yeast [18]. We therefore speculated that the *MRC1 *gene may be an excellent candidate for susceptibility to sarcoidosis, and tested for associations between *MRC1 *polymorphisms and the development of sarcoidosis in our Japanese case-control analysis.

## Methods

### Subjects

We enrolled unrelated subjects in this case-control study to search for susceptibility genes to sarcoidosis. A total of 181 Japanese subjects with sarcoidosis were recruited from the pulmonary clinic of the First Department of Medicine at Hokkaido University Hospital. Diagnosis of sarcoidosis in each patient was based on compatible clinical findings, histological demonstration of noncaseating epithelioid cell granuloma, and exclusion of other diseases capable of producing a similar histological or clinical picture, as recommended by the American Thoracic Society (ATS)/European Respiratory Society (ERS)/World Association of Sarcoidosis and Other Granulomatous Disorders (WASOG) statement. A total of 424 healthy controls, comprising individuals with no history of asthma or any other chronic pulmonary diseases, were recruited from individuals visiting the clinic for annual routine physical examinations and students at the School of Medicine at Hokkaido University. All subjects were unrelated and of Japanese descent. The medical ethics committee of Hokkaido University Graduate School of Medicine approved this study.

### SNP Selection and Genotyping

**Figure 1:**
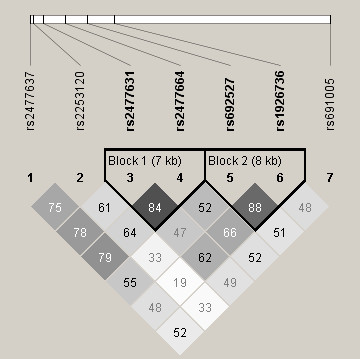
**Linkage disequilibrium structure (LD) of *MRC1 *gene across the 98.8 kb in Japanese (n = 605). **Black squares represent high pairwise linkage disequilibrium, coloring down to white squares of low pairwise linkage disequilibrium. The numbers in the individual squares are D' multiplied by 100.

**Figure 2:**
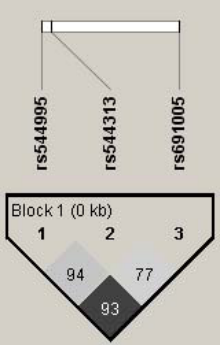
**Linkage disequilibrium structure (LD) of 3 SNPs (rs544995, rs544313 and rs691005) in *MRC1 *gene. **Black squares represent high pairwise linkage disequilib. The numbers in the individual squares are D' multiplied by 100.

Initially, we selected and genotyped seven SNPs in the *MRC1 *gene [12555A/G (rs2477637), 13789G/A (rs2253120), 17023A/C (rs2477631), 24351A/T (rs2477664), 31598A/G (rs692527), 40240G/A (rs1926736) and 111380T/C (rs691005)] based on our previous report [19]. As rs691005 showed a significant association, but major haplotyopes with strong linkage disequilibrium (LD) are not constructed by rs691005 using these 7 SNPs as described below (Figure 1), we genotyped two additional SNPs [11041G/A (rs554995) and 11065G/A (rs554313)] which are located close to rs691005. Additional LD structure using these 3 SNPs was shown in Figure 2. Alleles were identified using an assay combining kinetic (real-time quantitative) PCR with allele-specific amplification, as described elsewhere [19]. Real-time PCR was performed using SYBER Green I Master Mix (Applied Biosystems, Foster City, CA, USA) and an ABI PRISM TM 7700 Sequence Detection System (Applied Biosystems). Primers for allele specific PCR are shown in Table 1. rs554995 and rs554313 were genotyped using the TaqMan system (Applied Biosystems).

**Table 1:** Primers for allele specific PCR

Locus*	Primer sequence	
12555	Forward	5' ACTCAGTTACTTTCATTTGTTTATTCCTTAAC 3'
	Reverse for A	5' CCTTTAATTAAATCAAAATTGAGTTCAT 3'
	Reverse for G	5' CCTTTAATTAAATCAAAATTGAGTTCAC 3'
13789	Forward	5' GAATCTCAGATTATGAGTGTTGCATTT 3'
	Reverse for A	5' CATAGAGAGTGATAGCAACCCAGTCT 3'
	Reverse for G	5' CATAGAGAGTGATAGCAACCCAGTCC 3'
17023	Forward for A	5' GGGATTGCAAGCGTGAGACA 3'
	Forward for C	5' GGGATTGCAAGCGTGAGACC 3'
	Reverse	5' TTTGCAGATTCTACGACTTGAAAAAG 3'
24351	Forward for A	5' GAGCTCCTGAGCATCACAGAGATA 3'
	Forward for T	5' GAGCTCCTGAGCATCACAGAGATT 3'
	Reverse for A	5' ACTACCTGTCAGGTATGTTTGCTCAT 3'
	Reverse for T	5' CTTACCTGTCAGGTATGTTTGCTCAT 3'
31598	Forward for A	5' CAATAAAGGTCTCTGTTTAAAGTTTCAA 3'
	Forward for G	5' CAATAAAGGTCTCTGTTTAAAGTTTCAG 3'
	Reverse for A	5' CAACACATCAGGGATACTCTGAGAAT 3'
	Reverse for G	5' GTACCCAACACATCAGGGATACTCT 3'
40240	Forward	5' AGGGATGCTCTGACCACCTG 3'
	Reverse for A	5' GTGTGGATACTTGCGAGGTCTCT 3'
	Reverse for G	5' TGTGGATACTTGGGAGGTCTCC 3'
111380	Forward for C	5' TCTCTTTGGTACAACATAGTAAATCTCACC 3'
	Forward for T	5' TCTCTTTTTGGTACAACATAGTAAATCTCACT 3'
	Reverse for C	5' TTACCAACTGTTTTCCCATAATTGTG 3'
	Reverse for T	5' CCAACTGTTTTCCCATAATTGTGAG 3'

* Nucleotide numbering starts from the first nucleotide of the transcription start site.

### Statistical Analysis

Each of the SNPs in the *MRC1 *gene was tested for deviation from Hardy-Weinberg equilibrium using a χ^2 ^test. Both genotypic and allelic association among subjects with sarcoidosis and healthy controls were statistically compared using the logistic regression analysis adjusting for sex and age. The relative risk was estimated as odds ratios (OR) with 95% confidence intervals (95%CI). For LD mapping, pair-wise LD between polymorphisms in *MRC1 *was evaluated using Haploview software version 4.2 [20]. For haplotype analyses, we used the haplo.score program, which calculates simulation *P *values for each haplotype and further adjusts for covariates. Haplotypes with frequency below 5% were excluded from haplotype analysis. These statistical analyses were performed using a program R version 2.11.1 (http://www.R-project.org/) [21]. *P *values were adjusted using the Bonferroni correction for 10 tests. Levels of significance for all statistical analyses were set to *P *< 0.005.

## Results

Demographic characteristics of the 424 healthy controls and 181 subjects with sarcoidosis from this population are listed in Table 2. Median age was significantly higher for subjects with sarcoidosis than for healthy control subjects (*P *< 0.05). The sarcoidosis group included significantly more females than the control group (*P *< 0.05). Pair-wise LD values for 7 SNPs are shown in Figure 1. No significant deviation from the Hardy-Weinberg equilibrium was observed in healthy control subjects (*P *> 0.05). Genotype and allele frequencies and counts for each SNP in the *MRC1 *gene are shown in Table 3. When we performed logistic repression analysis using a recessive model adjusted for age and gender, 3 SNPs tended to be associated with sarcoidosis (rs2477637, OR 1.76, 95%CI, 1.09-2.84, *P *= 0.02; rs692527, OR 1.58, 95%CI 1.02-2.46, *P *= 0.042; and rs691005, OR 2.53, 95%CI 1.47-4.37, *P *= 0.001; Table 4). Additional association was observed for SNP rs2253120 (OR 1.62, 95%CI 1.12-2.36, *P *= 0.011), when analysis was performed under a dominant model. The rs691005 SNP remained statistically significant after Bonferroni correction. The linkage disequilibrium map constructed from 7 SNPs is shown in Figure 1, as measured by D prime and we identified two haplotype blocks. Haplotype block 1 comprised two SNPs (rs2477631 and rs2477664), and haplotype block 2 comprised two SNPs (rs692527 and rs1926736). However, significant association was not found in haplotype analysis (Table 5).

**Table 2:** Characteristics of 605 Japanese subjects

Japanese population	Control (n = 424)	Sarcoidosis (n = 181)	*P *value
Male (%)*	65.3	30.9	<0.05
**Age (median, range)**^**†**^	38, 18-72	45, 10-78	<0.05
**Stage (0/I/II/III/IV)**	-	21/90/59/11/0	
**Occular lesion (%)**	-	54.7	
**Cutaneous lesion (%)**	-	17.7	
**Cardiac lesion (%)**	-	7.2	

Data are presented as median (interquartile range). * χ^2 ^test † Mann-Whiteney U test

**Table 3:** Allele and genotype frequencies for 9 SNPs in *MRC1 *among subjects with sarcoidosis and control subjects

SNP ID	Locus*	Role	AA change	Allele	Controls n (%)	Cases n (%)	***P ***^**†**^	Genotype	Controls n (%)	Cases n (%)	***P ***^**‡**^
**rs2477637**	12555	intron1	none	A	547 (64.5)	213 (58.8)	0.062	AA	180 (42.5)	73 (40.3)	0.016
				G	301 (35.5)	149 (41.2)		AG	187 (44.1)	67 (37.0)	
								GG	57 (13.4)	41 (22.7)	
**rs2253120**	13789	exon2	none	G	638 (75.2)	253 (69.9)	0.053	GG	250 (59.0)	86 (47.5)	0.016
				A	210 (24.8)	109 (30.1)		AG	138 (32.5)	81 (44.8)	
								AA	36 (8.5)	14 (7.7)	
**rs2477631**	17023	intron2	none	A	443 (52.2)	191 (52.8)	0.868	AA	120 (28.3)	46 (25.4)	0.296
				C	405 (47.8)	171 (47.2)		AC	203 (47.9)	99 (54.7)	
								CC	101 (23.8)	36 (19.9)	
**rs2477664**	24351	exon4	none	A	455 (53.7)	191 (52.8)	0.776	AA	131 (30.9)	49 (27.1)	0.412
				T	393 (46.3)	171 (47.2)		AT	193 (45.5)	93 (51.4)	
								TT	100 (23.6)	39 (21.5)	
**rs692527**	31598	intron5	none	A	474 (55.9)	184 (50.8)	0.105	AA	130 (30.7)	50 (27.6)	0.145
				G	374 (44.1)	178 (49.2)		AG	214 (50.5)	84 (46.4)	
								GG	80 (18.9)	47 (26.0)	
**rs1926736**	40240	exon7	Gly/Ser	G	443 (52.2)	198 (54.7)	0.433	GG	111 (26.2)	56 (30.9)	0.455
				A	405 (47.8)	164 (45.3)		AG	221 (52.1)	86 (47.5)	
								AA	92 (21.7)	39 (21.5)	
**rs544995**	111041	3'-UTR	none	G	598 (70.5)	254 (70.2)	0.957	GG	200 (47.2)	88 (48.6)	0.526
				A	250 (29.5)	108 (29.8)		AG	198 (46.7)	78 (43.1)	
								AA	26 (6.1)	15 (8.3)	
**rs544995**	111065	3'-UTR	none	G	522 (63.4)	239 (66.0)	0.159	GG	163 (38.4)	75 (41.4)	0.148
				A	326 (39.6)	123 (34.0)		AG	196 (46.2)	89 (49.2)	
								AA	65 (15.3)	17 (9.4)	
**rs691005**	111380	3'-UTR	none	T	576 (67.9)	226 (62.4)	0.064	TT	191 (45.0)	79 (43.6)	0.003
				C	272 (32.1)	136 (37.6)		TC	194 (45.8)	68 (37.6)	
								CC	39 (9.2)	34 (18.8)	

* Nucleotide numbering starts from the first nucleotide of the transcription start site.

† Allele frequencies of each SNP were compared between sarcoidosis and controls by χ2 test (2 × 2).

‡ Overall genotype differeneces were compared between sarcoidosis and controls by χ2 test (2 × 3).

**Table 4:** Odds ratios (OR) and *P *values for 9 SNPs in *MRC1 *among subjects with sarcoidosis and control subjects

	Dominant model		Recessive model	
	OR [95% CI]*	*P *value*	OR [95% CI]*	*P *value*
**rs2477637**	1.04 [0.71-1.52]	0.843	1.76 [1.09-2.84]	0.02
**rs2253120**	1.62 [1.12-2.36]	0.011	0.83 [0.42-1.65]	0.593
**rs2477631**	1.08 [0.70-1.64]	0.739	0.79 [0.50-1.24]	0.298
**rs2477664**	1.18 [0.78-1.78]	0.433	0.87 [0.56-1.36]	0.535
**rs692527**	1.15 [0.76-1.74]	0.496	1.58 [1.02-2.46]	0.042
**rs1926736**	0.78 [0.52-1.17]	0.222	0.97 [0.62-1.53]	0.906
**rs554995**	0.90 [0.62-1.31]	0.588	1.43 [0.70-2.95]	0.326
**rs554313**	0.83 [0.57-1..21]	0.336	0.65 [0.36-1.18]	0.159
**rs691005**	1.00 [0.69-1.46]	0.987	2.53 [1.47-4.37]	0.001

* logistic regression model: adjusted by sex and age

**Table 5:** Estimated haplotype frequencies and association with sarcoidosis

Haplotype	controls (%)*	cases (%)*	total (%)*	haplotype-specific score	**simulated *P***^†^
**rs2477631-rs2477664**					
A-A	48.6	48.4	48.6	-0.171	0.863
C-T	42.7	42.9	42.8	0.089	0.929

**rs692527-rs1926736**					
A-A	46.0	41.4	44.6	-1.601	0.111
A-G	9.8	9.5	9.7	-0.428	0.667
G-G	42.4	45.2	43.2	1.074	0.279

**rs554995-rs554313-rs691005**					
G-G-T	30.6	30.6	30.6	0.098	0.916
A-G-C	27.7	29.4	28.1	0.741	0.452
G-A-T	29.3	36.2	34.7	2.220	0.028

*Minimum haplortpe frequency was 0.05 for which haplotypes were scored in the model.

†adjusted by sex and age

Based on LD structure and significant association of rs691005 for initial analysis, we further genotyped two additional SNPs (rs554995 and rs554313) close to rs691005. Unfortunately, these two SNPs did not show significant associations (Tables 3 and 4). Although three SNPs (rs554995, rs554313 and rs691005) showed strong LD, significant associations were not found in haplotype analysis (Table 5).

## Discussion

In the present study we demonstrated the association between *MRC1 *polymorphisms and risk of sarcoidosis in Japanese population. In support of this, the association of one SNP (rs691005) was confirmed considering for multiple testing and Bonferroni correction, suggesting *MRC1 *gene as a plausible candidate gene for development of sarcoidosis. Of interest, recent genome-wide association analyses have shown that 10p12, where *MRC*1 is situated, is a susceptibility locus for the development of sarcoidosis [22]. Thus, findings of the current study suggest that *MRC1 *gene variants may contribute to the development of sarcoidosis.

The rs691005 located within the 3'-untranslated region (3'-UTR) of *MRC1 *showed the strongest association (OR 2.53). Although the real functions of this gene are unclear, variants in the 3'-UTR are known to disrupt a regulatory binding sequence and alter mRNA expression [23]. Alternatively, this variant may be representative of the region or correlated with a true functional variant. Thus, our current results provide a basis for further identification of the causative variants underlying the relationship between *MRC1 *gene sequences and sarcoidosis.

It should be noted that SNPs with two positions were mapped to *MRC1 *spanning chr10:17,891,368-17,993,183 (HapMap Data Rel 27) and were referred to by their *MRC1L1 *'rs'numbers (NCBI EntrezSNP database Build 130). Alter et al reported no evidence for a common gene duplication event [24]. The authors suggested that *MRC1L *is an erroneous annotation caused by the presence of a sequence gap and the incorrect assignment of a polymorphic haplotype.

We also reported the association of *MRC1 *gene polymorphism and risk of asthma in two independent ethnically diverse populations [19], suggesting that *MRC1 *might be involved in the pathogenesis of a number of chronic inflammatory diseases. Several reports have shown that genetic variants of genes related to PRRs such as TLR4 and CD14 are associated with susceptibility to both diseases [10,11,25-27]. The present study adds further evidence supporting the involvement of macrophage PRRs in the development of sarcoidosis as a chronic inflammatory lung disease.

Of the SNPs examined, three SNPs (rs26777637, rs2253120 and rs692427) showed tendency for association with sarcoidosis (*P *= 0.02, *P *= 0.011, *P *= 0.042), but this association did not reach significance after the Bonferroni correction. However, associations of rs691005 remained significant even after Bonferroni correction (*P *= 0.001). In addition, power calculations based on study subjects of 181 cases and 424 controls, OR of 2.53 showed a sufficient genetic power (0.81) at the level of significance of 0.005. As the sample size of this study is not sufficiently large and is restricted to Japanese population, the present data should be validated in larger samples and in other ethnic groups.

## Conclusions

This study suggests that the *MRC1 *gene may represent an important susceptibility locus for sarcoidosis at chromosome 10p12 and genetic variants in *MRC1 *may play significant roles in the pathogenesis of sarcoidosis. Importantly, the association we observed between *MRC1 *polymorphisms and sarcoidosis adds further evidence for the involvement of macrophage PRRs in the development of a number of chronic inflammatory diseases, including sarcoidosis. However, further studies are clearly needed to achieve a comprehensive coverage of genetic variants in and around the *MRC1 *gene, in order to identify causal variants conferring susceptibility to an increased risk of sarcoidosis.

## Competing interests

The authors declare that they have no competing interests.

## Authors' contributions

The authors TH, SK, PG, SH, NH and MN made substantial contribution to the conception and design of the study, and analysis and interpretation of the data. AT, AI, Kaouruko S, Kenichi S, and NT, and EY made a substantial contribution to the collection of the resources and an intellectual contribution to the study design. All authors read and approved the final version.

## Pre-publication history

The pre-publication history for this paper can be accessed here:

http://www.biomedcentral.com/1471-2350/11/151/prepub
